# Impact of Corticosterone Treatment on Spontaneous Seizure Frequency and Epileptiform Activity in Mice with Chronic Epilepsy

**DOI:** 10.1371/journal.pone.0046044

**Published:** 2012-09-28

**Authors:** Olagide W. Castro, Victor R. Santos, Raymund Y. K. Pun, Jessica M. McKlveen, Matthew Batie, Katherine D. Holland, Margaret Gardner, Norberto Garcia-Cairasco, James P. Herman, Steve C. Danzer

**Affiliations:** 1 Department of Physiology, Ribeirão Preto School of Medicine, University of São Paulo, Ribeirão Preto, São Paulo, Brazil; 2 Program in Neuroscience, University of Cincinnati, Cincinnati, Ohio, United States of America; 3 Division of Clinical Engineering, Cincinnati Children's Hospital Medical Center, Cincinnati, Ohio, United States of America; 4 Division of Neurology, Cincinnati Children's Hospital Medical Center, Cincinnati, Ohio, United States of America; 5 Department of Anesthesia, Cincinnati Children's Hospital Medical Center, Cincinnati, Ohio, United States of America; 6 Molecular and Developmental Biology Program, University of Cincinnati, Cincinnati, Ohio, United States of America; 7 Department of Psychiatry and Behavioral Neuroscience, University of Cincinnati, Cincinnati, Ohio, United States of America; 8 Departments of Anesthesia and Pediatrics, University of Cincinnati, Cincinnati, Ohio, United States of America; Beijing Institute of Radiation Medicine, China

## Abstract

Stress is the most commonly reported precipitating factor for seizures in patients with epilepsy. Despite compelling anecdotal evidence for stress-induced seizures, animal models of the phenomena are sparse and possible mechanisms are unclear. Here, we tested the hypothesis that increased levels of the stress-associated hormone corticosterone (CORT) would increase epileptiform activity and spontaneous seizure frequency in mice rendered epileptic following pilocarpine-induced status epilepticus. We monitored video-EEG activity in pilocarpine-treated mice 24/7 for a period of four or more weeks, during which animals were serially treated with CORT or vehicle. CORT increased the frequency and duration of epileptiform events within the first 24 hours of treatment, and this effect persisted for up to two weeks following termination of CORT injections. Interestingly, vehicle injection produced a transient spike in CORT levels – presumably due to the stress of injection – and a modest but significant increase in epileptiform activity. Neither CORT nor vehicle treatment significantly altered seizure frequency; although a small subset of animals did appear responsive. Taken together, our findings indicate that treatment of epileptic animals with exogenous CORT designed to mimic chronic stress can induce a persistent increase in interictal epileptiform activity.

## Introduction

Stress is widely recognized as a precipitating factor for seizures in many patients with epilepsy [1]–[4]. Despite a clear correlation between stress and seizures such that many patients are able to predict their own seizures with great accuracy [5]–[7], whether a causal relationship between stress and epilepsy exists is not known. Indeed, changes in the brain preceding a seizure might increase patient perceptions of stress and anxiety rather than the converse. Alternatively, factors related to stress, such as sleep loss, rather than stress *per se* may account for the correlation. Nonetheless, understanding the relationship between stress and seizures is important. Chronic stress can lead to lasting changes in brain structure and function [8]–[11], providing a possible mechanism by which stress might impact epilepsy. For example, in the hippocampus – a brain region implicated in temporal lobe epilepsy – stress alters dentate granule cell proliferation [12], [13] and regulates dendritic and synaptic plasticity [14]–[16]. Therefore, it is conceivable that stressful events could increase the likelihood that at-risk individuals will develop epilepsy, or worsen the course of the disease in patients with pre-existing epilepsy. Identifying such “disease-modifying” effects of stress on epilepsy could be extremely important for clinical management of the disorder.

Stress activates the hypothalamic–pituitary–adrenal (HPA) axis, leading to the release of a variety of neuropeptides and hormones [17]. Glucocorticoids – corticosterone (CORT) in rodents and cortisol in humans – are prominent among these. CORT exerts its effects through high-affinity mineralocorticoid receptors and comparatively lower affinity glucocorticoid receptors. Both receptors act by binding DNA response elements to alter gene expression, and are thus capable of producing relatively slow developing but long-lasting changes in transcription. In addition, glucocorticoids can also act through non-genomic mechanisms, leading to more rapid changes in behavior and physiology [18], [19]. Both receptor types are expressed at high levels in cortex and hippocampus [20]–[24] and act to regulate the excitability of cortical and hippocampal neurons [25].

Although the mechanisms underlying epileptogenesis are still being elucidated, it is clear that many patients' seizures originate from discrete brain regions. These brain regions often exhibit damage from injury (e.g. hypoxia, status epilepticus), tumors or developmental defects (e.g. heterotopias), and are commonly found in the cortex or temporal lobe. Surgical removal of these damaged regions is highly effective at controlling seizures [26], providing compelling evidence that these regions originate the seizures. Evidence for disease-causing brain lesions in patients with epilepsy, however, fails to explain the paroxysmal nature of the disorder. To restate the question, why should epileptic foci, which are always present, only occasionally provoke seizures? The implication is that most of the time, the balance between excitation and inhibition in the brain is adequately maintained such that despite the presence of an epileptic focus, seizures do not occur. Changes in factors which can alter excitation and inhibition in brain presumably upset this balance sporadically, provoking seizures. The present study explores whether CORT could be one of those factors.

To determine whether increasing CORT levels in animals with pre-existing epilepsy would provoke seizures, we treated epileptic mice with exogenous CORT while monitoring ictal and interictal EEG activity. Animals were rendered epileptic using the pilocarpine-status epilepticus model of epilepsy.

## Results

**Figure 1:**
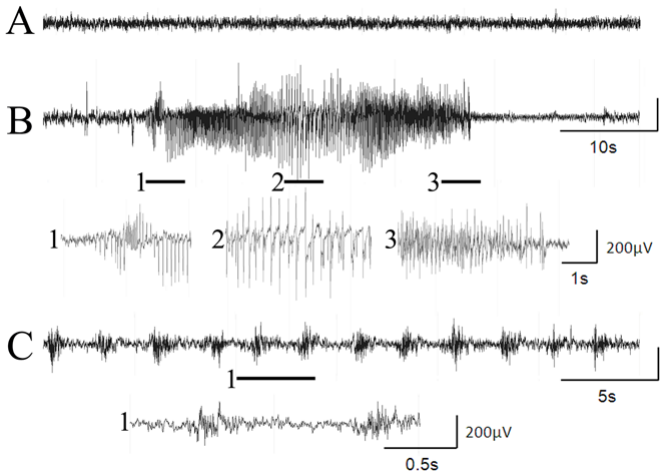
EEG recordings from epileptic animals. **A:** Baseline EEG activity. **B:** A typical seizure event in an epileptic animal. This seizure was associated with behavioral rearing and loss of postural control (falling). **C:** An example of epileptiform activity recorded from an epileptic animal. The EEG segments shown below B and C are expansions of the periods marked by the solid bars. Animals were typically motionless or exhibited myoclonic jerks during epileptiform events.

In the present study, epileptic animals were implanted with wireless EEG transmitters and were monitored by video-EEG 24/7 to determine the impact of corticosterone (CORT) treatment on epileptic activity. EEG records were screened to identify epileptiform events and overt seizures. EEG seizures were characterized by the sudden onset of high amplitude activity, with evolving frequency and amplitude over the course of the event, and abrupt termination (Fig. 1, B). Seizures were frequently, but not always, associated with behavioral symptoms that included mouth automations, myoclonic jerking, forelimb clonus, rearing and falling (up to class V, Racine scale) [27]. By contrast, epileptiform activity was defined here as periods of repetitive spiking or bursting with a frequency of 3–120/minute and lasting for at least 30 seconds (Fig. 1, C). This activity was similar to patterns described in other models of epilepsy [28], and tended to wax and wane over time. During epileptiform events, animals typically exhibited little or no movement, and did not engage in normal behaviors (exploring the cage, eating, grooming) although they remained upright. Cessation of epileptiform activity corresponded with a return to normal behavior.

### Corticosterone increases epileptiform activity

**Figure 2:**
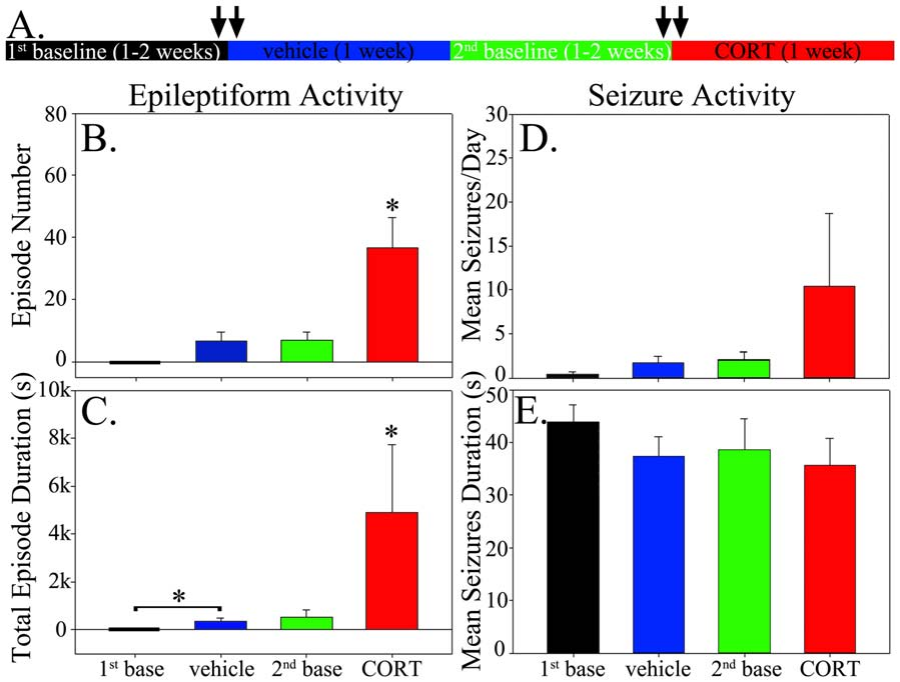
Schematic and graphs illustrating the experimental approach (A) and results (B–E) for animals treated with vehicle followed by CORT. **A:** Experimental protocol for the vehicle-first treatment regimen. Video-EEG was monitored continuously throughout the entire regimen, with each period corresponding to 1–2 weeks. Twenty-four hour EEG segments quantified for epileptiform activity are denoted by arrows. The color of each part of the timeline corresponds to the bar color in the graphs shown below. **B:** CORT treatment significantly increased the number of epileptiform events relative to all other periods. **C:** CORT treatment significantly increased the total duration of epileptiform events relative to all other periods. Vehicle treatment also significantly increased the duration of events relative to the 1^st^ baseline period. **D:** No significant effects of vehicle or CORT treatment on seizure frequency were found. **E:** Mean seizure duration was statistically equivalent among treatment groups and baseline periods. Bar graphs show means ± SEM. *, p<0.05.

To determine whether CORT altered the incidence of epileptiform activity in epileptic animals, epileptiform events were quantified during the 24-hour periods immediately preceding and following the first vehicle and CORT injections (Fig. 2, A, arrows). CORT significantly increased the number (p<0.032) and total duration (p<0.047) of epileptiform EEG events in epileptic animals relative to baseline EEG activity in the preceding 24 hours (Fig. 2, B–C). Vehicle injection also significantly increased the duration (p = 0.049) of epileptiform events relative to baseline (Fig. 2, C). The response to vehicle treatment, however, was significantly less than that following CORT treatment (p = 0.017 for event number; p = 0.022 for duration). Epileptiform activity was absent from control animals under all conditions.

### Effects of corticosterone treatment on seizure frequency

**Figure 3:**
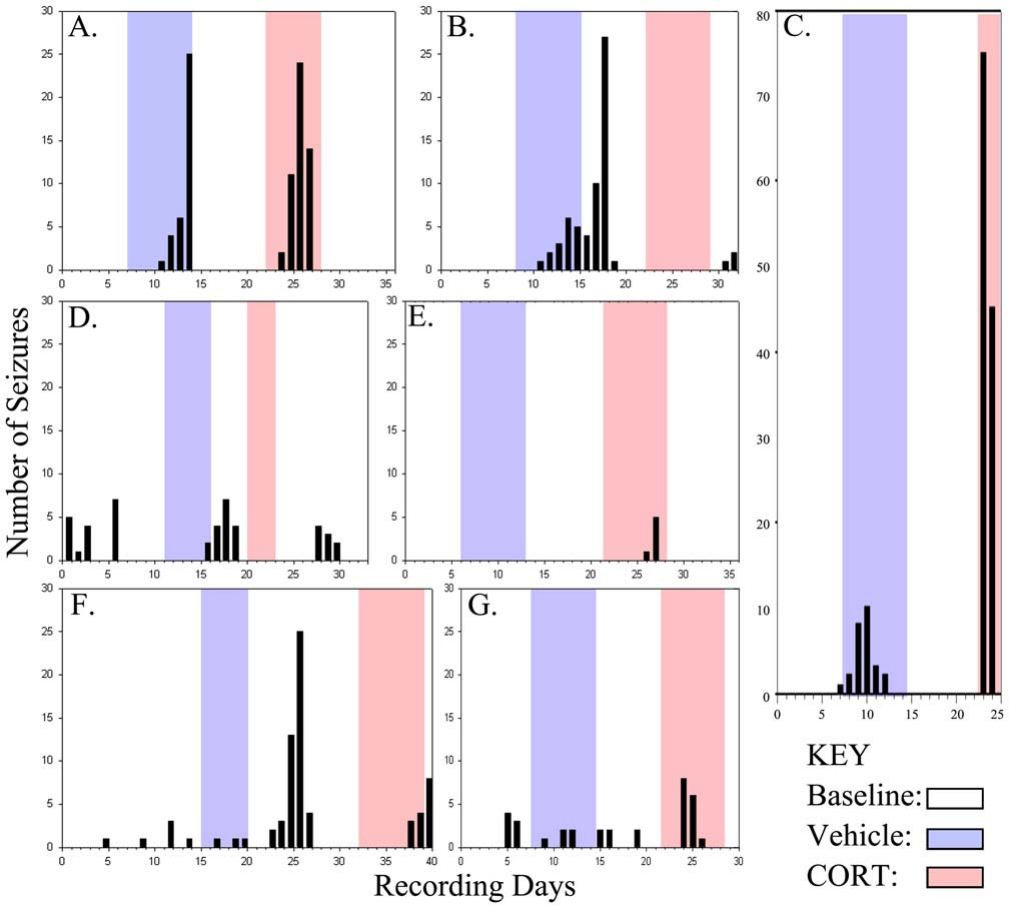
Individual seizure timelines of epileptic animals receiving vehicle first. Periods of vehicle and CORT treatment are highlighted in blue and red, respectively, while baseline periods are left white. Bars denote the number of seizures on a given day. One animal exhibited seizures only during CORT treatment (E), while 2 animals (A & C) exhibited seizures primarily during periods of vehicle and CORT treatment. The remaining four animals showed no compelling correlation between seizure incidence and treatment, and overall there was no significant effect of treatment on seizure frequency.

To determine whether CORT treatment altered seizure frequency or duration, seizure rates during vehicle treatment and CORT treatment periods were compared to rates during all other periods. No significant changes in seizure frequency (Fig. 2, D; p = 0.447) or mean seizure duration (Fig. 2, E; p = 0.294, Kruskal-Wallis One Way ANOVA on Ranks) were found among the different treatments. Although overall rates were not significantly different for the entire group of animals, one animal exhibited seizures only during CORT treatment (Fig. 3, E) and two animals appeared to respond to both vehicle and CORT treatment with increased seizures (Fig. 3, A & C). Indeed, the animal shown in C exhibited a dramatic increase in seizures during CORT treatment on the first day and died on the second day after having 45 seizures. Whether the apparent responses to CORT among a subset of animals reflect actual responses, or just coincidence, however, remains uncertain. None of the control animals showed any baseline seizures nor did they exhibit any seizures during vehicle or CORT injections (data not shown).

### Measurement of corticosterone levels in plasma

**Figure 4:**
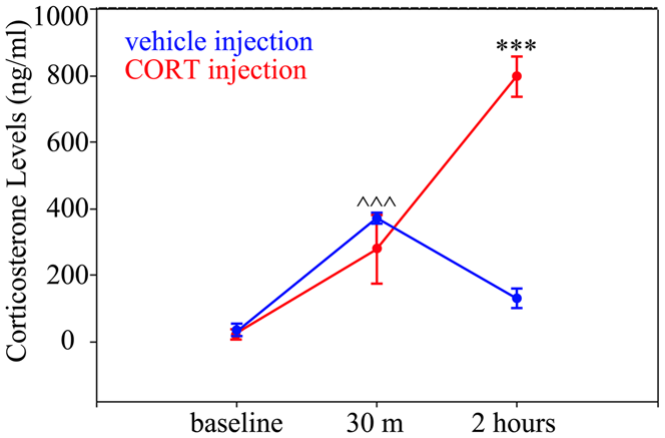
Plasma corticosterone levels measured in a group of non-epileptic (control) mice. CORT levels were determined following vehicle (blue) and CORT (red) injection. Levels were measured just prior to injection (baseline), thirty minutes (30 m) after injection and two hours after injection. At 30 minutes, both vehicle and CORT injection elevated CORT levels significantly. At 120 minutes, CORT levels began to come down in the case of the vehicle injection, though still significantly higher than baseline levels. Following CORT injection, levels continued to increase beyond 30 minutes. ? ? ?, p<0.001 vs. baseline for both vehicle and CORT; ***, p<0.001 vs. all other groups and time points.

In the present study, animals were treated sequentially with vehicle for one week, followed by a 1–2 week recovery and then CORT for one week. To confirm that this treatment paradigm was effective at raising CORT above baseline levels, four control mice were run through the protocol. On the first day of vehicle or CORT treatment, CORT levels were measured just prior to injection, as well as thirty minutes and two hours after injection. Thirty minutes after vehicle and CORT injections, CORT levels were increased 10–12 fold relative to the respective baseline (p<0.001; two-way ANOVA on ranked data with treatment and time after injection as factors; Holm-Sidak post-test). The increase following vehicle injection likely reflects endogenous CORT due to handling stress [29]. Two hours after injections, CORT levels began to decline in vehicle treated animals (2 hrs vs. 30 m, p = 0.009), though levels were still significantly above baseline (2 hrs vs. baseline, p = 0.010). In CORT treated animals, CORT levels increased dramatically between thirty minutes and two hours (p<0.001). At two hours, CORT levels in CORT injected animals were significantly higher than vehicle injected animals at the same time point (p<0.001). Plasma CORT levels for these time points are shown in figure 4. Our results indicate that vehicle injection can lead to a stress induced increase in CORT, however, the elevation of CORT is transient and of lower amplitude than levels produced by CORT injection.

### CORT effects persist beyond the termination of treatment

Given the transient but significant increase in CORT from vehicle injection alone, and increased duration of epileptiform activity in vehicle injected mice (Fig. 2, C), we queried whether epileptic animals might respond differently if the protocol was reversed and “naïve” epileptic animals were given CORT in the first injection.

**Figure 5:**
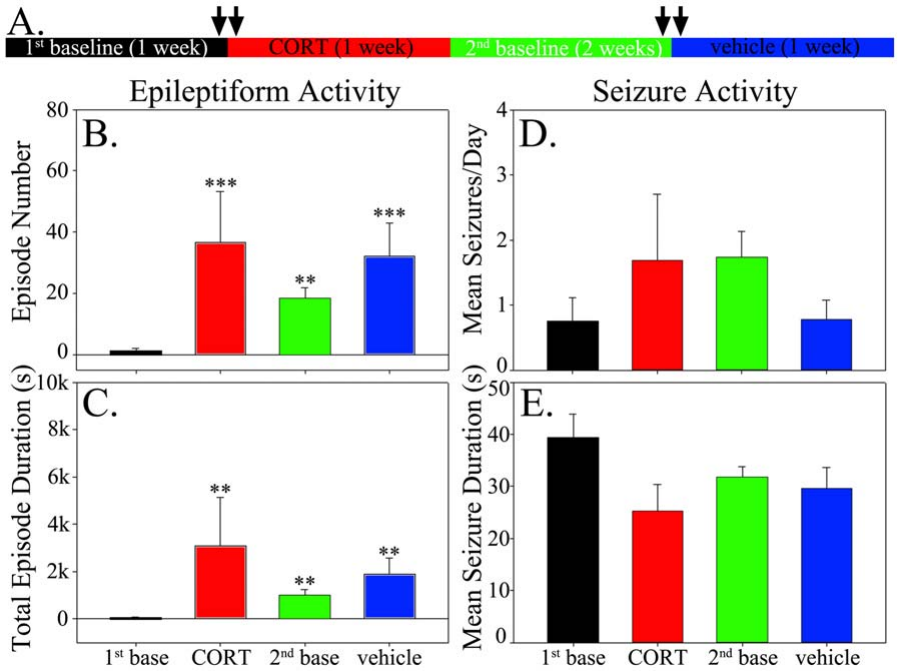
Schematic and graphs illustrating the experimental approach (A) and results (B–E) for animals treated with CORT followed by vehicle. **A:** Experimental protocol for the CORT-first treatment regimen. Video-EEG was monitored continuously throughout the entire regimen, with each period corresponding to 1–2 weeks. Twenty-four hour EEG segments quantified for epileptiform activity are denoted by arrows. The color of each part of the timeline corresponds to the bar color in the graphs shown below. **B:** CORT treatment significantly increased the number of epileptiform events relative to the preceding baseline period (1^st^ base). The number of epileptiform events was also increased during subsequent periods (2^nd^ baseline, vehicle) relative to the first baseline. **C:** CORT treatment significantly increased the total duration of epileptiform events relative to the preceding baseline period. The total duration of epileptiform events was also increased during subsequent periods relative to the first baseline. **, p<0.01 vs. 1^st^ baseline; ***, p<0.001 vs. 1^st^ baseline. **D:** No significant effects of vehicle or CORT treatment on seizure frequency were found. **E:** Mean seizure duration was statistically equivalent among treatment groups and baseline periods. Bar graphs show means ± SEM.

**Figure 6:**
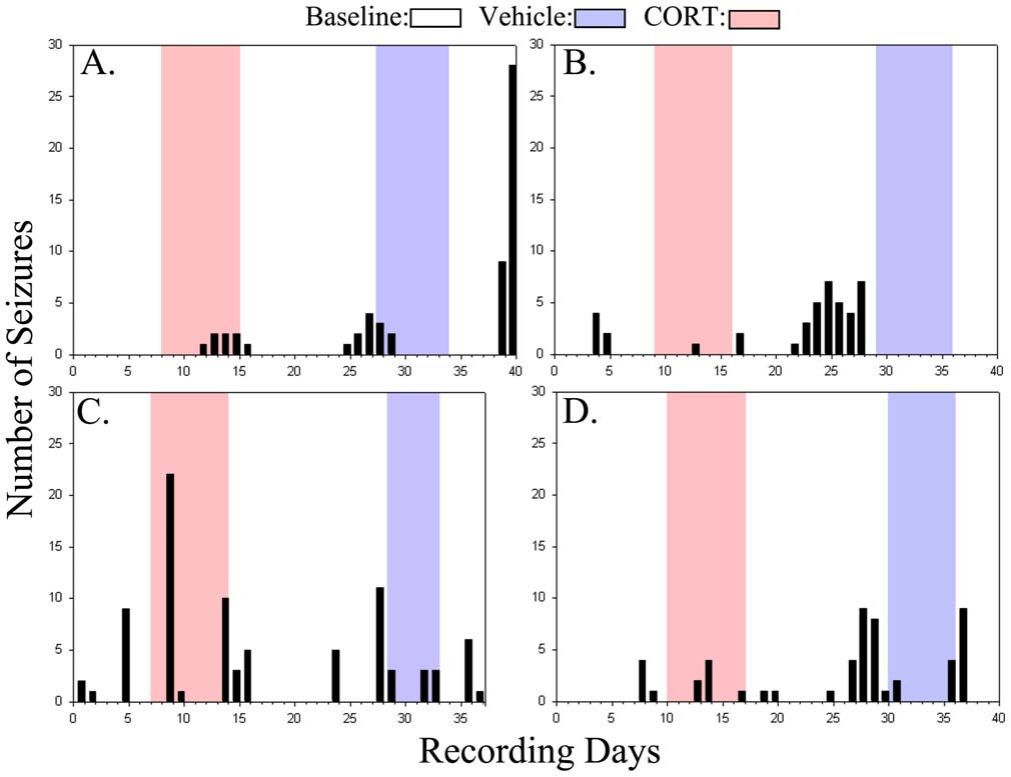
Individual seizure timelines of epileptic animals receiving CORT first. Periods of vehicle and CORT treatment are highlighted in blue and red, respectively, while baseline periods are left white. Bars denote the number of seizures on a given day. No striking correlations between treatment and seizure incidence were observed for these animals.

Despite the reversed protocol, CORT treatment did not significantly alter seizure frequency (Fig. 5, D; p = 0.273) or duration (Fig. 5, E; p = 0.175), suggesting that prior vehicle injection stress did not blunt the response to exogenous CORT. Seizure patterns within animals also failed to exhibit any compelling correlations between treatment periods and seizure incidence (Fig. 6). Consistent with the findings from animals treated with vehicle first, however, CORT still produced a dramatic increase in the number (p<0.001) and total duration (p = 0.002) of epileptiform events relative to the preceding baseline period (Fig. 5, B–C).

The reversed protocol revealed one additional surprising finding. Specifically, once epileptic animals had been exposed to CORT, the number and duration of epileptiform events never recovered to pre-CORT baseline levels. Relative to the first baseline, both the number (p = 0.003) and duration (p = 0.003) of epileptiform events was increased during the second (post-CORT) baseline. Similar increases were observed during vehicle treatment relative to the first baseline (Fig. 5, B–C). The baseline period following CORT treatment ranged from 12–14 days, and epileptiform events were quantified during the last 24 hours of this period, so this increase was evident despite an almost two-week recovery period since the last CORT injection. Moreover, this would not appear to be due to epilepsy progression in these animals unrelated to CORT treatment, because animals in the first experiment did not exhibit an increase in epileptiform activity during their second baseline monitoring period (Fig. 2, B–C), despite being at equivalent time points after status epilepticus (vehicle first group, 3.4±1.9 months after SE at beginning of protocol; CORT first group, 3.6±2.6 months; p = 0.939, t-test). A post-hoc comparison for the 2^nd^ baseline period between the vehicle-first group and the CORT-first group revealed that the latter was significantly greater than the former for both the number (p = 0.004, t-test) and duration (p = 0.003, t-test) of epileptiform events. These findings strongly suggest that exposure to exogenous CORT, and not time after status epilepticus, is responsible for the persistent increase in epileptiform activity.

### Seizure activity does not exhibit diurnal rhythms

Corticosteroid levels show diurnal variations in rodents; levels are low in the morning and peak in the evening. To explore whether these variations in endogenous CORT levels might impact excitability, seizure activity during the first week of recording (before any treatments) was binned into four 6-hour segments. For the six animals that had seizures during the baseline period, no significant variations in seizure frequency among the four periods were found (6AM-Noon, 0.20±0.14 seizures/6 hr period; Noon-6PM, 0.39±0.16; 6PM-Midnight, 0.44±0.23; Midnight-6AM, 0.25±0.13; one-way ANOVA, p = 0.711), consistent with recent work [30]. Since animals did not exhibit epileptiform activity (as defined in the methods) during the first week of recording, diurnal variations were not examined for this parameter.

## Discussion

Stress is the most commonly reported seizure precipitant in patients with epilepsy [1]–[4]. A mechanistic link between stress and seizures, however, is lacking. Here, we sought to determine whether exogenous CORT treatment at levels designed to mimic chronic stress would provoke seizures in epileptic animals. CORT is increased under stressful conditions [31] and since CORT can enhance neuronal excitability [25], it is an obvious candidate for modulating seizure frequency in patients with epilepsy. Interestingly, while we did not find a significant increase in overt seizures following CORT treatment, CORT produced a robust and long-lasting increase in epileptiform activity. Epileptiform events were characterized by repetitive spiking and/or bursting in the cortical EEG. These events could last for minutes to hours during which the animals were largely immobile. The increase in epileptiform events persisted for at least two weeks after the last CORT injection, indicating that CORT can have long-lasting effects on excitability in epilepsy. Vehicle injection also increased the duration of epileptiform events, although effects were modest relative to CORT and did not persist. Perhaps not coincidentally, vehicle injection produced a transient increase in plasma CORT levels. It is conceivable that this endogenous CORT, induced by injection stress, accounts for the increase in epileptiform activity. Importantly, none of the control, non-epileptic animals exhibited epileptiform activity or seizures in their EEG following vehicle or CORT administration. Taken together, our results support the conclusion that CORT can induce a long-lasting increase in epileptiform activity that is specific to epileptic animals. These findings further suggest that CORT could have disease-modifying effects in epilepsy.

### Relevance to prior studies

Numerous studies have demonstrated that CORT treatment can alter neuronal activity in normal animals. For example, CORT can increase the frequency of miniature excitatory postsynaptic currents [32], enhance L-type calcium currents [33] and paradoxically, can both facilitate and impair the induction of long-term potentiation (LTP), depending on the timing of treatments and presence of other neuromodulators [34]–[36]. The effects of CORT on LTP highlight the complex dynamics underlying CORT responses. Indeed, the impact of CORT can vary among brain regions and neuronal subtypes. It can act synergistically or antagonistically with other hormones and an animal's response to CORT can be influenced by its life history (e.g. perinatal stress exposure). Notably, all of these studies were conducted in normal animals and CORT effects on the epileptic brain may differ significantly.

The existing literature provides some additional insight into the effects of CORT on the epileptic brain. Cortisone treatment of photosensitive baboons has been shown to increase seizure activity [37] and corticosterone treatment of WAG/Rij rats, used to model absence epilepsy, increased the incidence of spike-wave discharges [38]. Stress also increased spike-wave discharges in these animals [39], a finding also reminiscent of the present work. For these latter studies, however, we note that the mechanisms underlying absence epilepsy are thought to be very different than those underlying the temporal lobe epilepsy model used here, so the comparison is made cautiously. Stressor exposure has been shown to provoke seizures in a number of epilepsy models [40], [41], although whether this effect is mediated by CORT is not known. A greater number of studies have demonstrated pro-convulsant effects of CORT under acute conditions in normal animals, although these findings again reflect the actions of CORT on the normal, not epileptic, brain. Nonetheless, it has been demonstrated that CORT can act synergistically with the convulsant drug kainic acid. Specifically, CORT decreases the dose of kainic acid required to produce seizures in mice [42], and increases the number of brain slices exhibiting multiple population spikes in response to Schaeffer collateral stimulation, a measure of epileptogenesis [43]. In the kindling model of epilepsy, CORT has been found to reduce the number of stimulations required to evoke seizures, significantly accelerating kindling epileptogenesis [44]–[47]. Whether CORT exacerbates or mitigates neuronal injury in seizure models is controversial, with studies demonstrating both protective [48] and neurotoxic effects [49]–[51]. Differences in treatments and their timing, species and models are likely important, and again highlight the complexity of this system [52]. In general, however, the majority of studies support the idea that CORT can act in a pro-convulsant, and possibly pro-epileptogenic fashion.

### Seizure frequency in epileptic animals following CORT treatment

Although CORT clearly increased epileptiform activity in epileptic animals, no change in seizure frequency was found. Nevertheless, we interpret this negative result somewhat cautiously. Specifically, although there was no overall effect on seizure frequency, three of the ten animals (Fig. 3, A, C & E) exhibited a striking correspondence between treatments and seizure incidence, with seizures occurring primarily during periods of vehicle or CORT injection, and with the latter being associated with the greater number of seizures. Since vehicle injection transiently increased CORT levels, CORT could be provoking seizures under both treatment conditions. It is possible that not all epileptic animals will respond to CORT with increased seizures, so the apparent lack of response in some animals does not preclude the possibility of a response in others. Indeed, studies of human epilepsy clearly indicate that some patients report stress-induced seizures, and some do not [4]. It is reasonable to think that animals might show similar variability, and although attempts were made to keep experimental animals as homogenous as possible, the pilocarpine model of epilepsy can produce very heterogeneous patterns of cell loss and plasticity [53]. No overt differences in gross brain pathology were observed between “responding” and “non-responding” animals in the present study (data not shown), but the possibility that more subtle differences are important cannot be excluded.

A second feature that might make it difficult to detect an effect of CORT on seizure frequency is the tendency for seizures to occur in clusters. Seizure clustering has been observed previously [54]–[57], and may reflect excitatory changes in the pre-ictal period that make seizures more likely, followed by a refractory period during which seizures are inhibited. In the present study, group assignments were made randomly, and no attempt was made to coordinate seizure clusters with treatments to avoid biasing the results; however, the timing of CORT treatments relative to the stage of this natural cycle could exert an important modulatory effect on the CORT response. This interesting question could be addressed with even longer recording periods and a systematic timing of CORT injections, although such experiments will require improved technologies, as transmitter battery life is only 8–10 weeks. Alternatively, other animal models of epilepsy with more constant daily seizure rates might be superior for assessing the impact of CORT on epilepsy.

Variability in endogenous CORT levels and HPA axis function might also impact the response of epileptic mice to exogenous CORT. Seizures increase CORT levels acutely [58], [59], and epileptic animals [60], [61] and humans [62] exhibit hyperactive HPA axes. Complex interactions between exogenously applied CORT and endogenous CORT rhythms could make it more difficult to identify a clear effect of treatment. Disrupted HPA axis function in epileptic animals could also make it more difficult to identify any effects of endogenous CORT rhythms on seizure occurrence. We examined seizure occurrence during the baseline monitoring period – prior to any treatments the might alter the diurnal CORT rhythm – and did not observe any variation in seizure incidence throughout the day. Similar results have been reported previously [30], however, the extent to which epileptic animals follow the diurnal CORT rhythms seen in healthy controls is not clear, so negative results should be interpreted with this caveat in mind. Future studies using methods to suppress the effects of endogenous CORT, such as adrenalectomy, glucocorticoid antagonists and/or genetic strategies [63], might prove superior at exploring the possible role of endogenous CORT in seizure occurrence.

### Significance of increased epileptiform activity

The major finding of the present study is that CORT treatment increases epileptiform activity in animals with pre-existing epilepsy, and that this increase persists for at least two weeks after the last CORT injection. Epileptiform activity is associated with greater cognitive disruption and poorer prognosis in patients with epilepsy [64]–[66], so the changes observed here are likely harmful. Furthermore, this finding is significant as it suggests that CORT may exert disease-modifying effects on the epileptic brain. In contrast to agents which only affect the disease phenotype when present, a disease-modifying agent could produce a lasting, and potentially irreversible, change in disease severity or progression. Whether the changes observed here persist beyond two weeks is not known, but the present findings suggest that levels of CORT similar to those observed during chronic stress might produce a lasting increase in abnormal EEG activity in patients with epilepsy.

## Materials and Methods

### Pilocarpine treatment

All procedures involving animals were approved by the Institutional Animal Care and Use Committee of the Cincinnati Children's Hospital Research Foundation and conform to National Institute of Health guidelines for the care and use of animals. Four to six-week-old male C57BL/6 mice (Charles River Laboratories International, Inc., Wilmington, MA) were housed on a 14/10 (light/dark) cycle with access to food and water *ad libitum*. Between 8–10 weeks of age, the animals were pre-treated with a subcutaneous (s.c.) injection of methyl scopolamine nitrate (1 mg/kg) followed 15 minutes later by 420 mg/kg pilocarpine s.c. All pilocarpine treatments were done between 10:00 AM and 12:00 PM to limit diurnal variation. Mice were observed to determine the onset of status epilepticus (SE), defined behaviorally by continuous tonic/clonic convulsions or shorter tonic/clonic convulsions without recovery between episodes. Three hours after the onset of SE, mice received two doses of diazepam (10 mg/kg; Sigma-Aldrich, St. Louis, MO) at 15 min intervals. Mice were housed in a 32°C incubator for the next 48 hours and given warm (30–33°C) Ringers s.c. (3–5 times) to maintain pretreatment body weight and improve recovery.

### 24-7 Video-EEG monitoring

Eleven pilocarpine-treated epileptic animals were implanted with wireless transmitters for continuous EEG monitoring [67]–[70]. Animals were implanted 1–12 months after pilocarpine-status epilepticus. In addition, five untreated control mice were also implanted. For transmitter implantation, animals were anesthetized with 3.5% isoflurane in oxygen and then maintained at 0.7–1.2% isoflurane throughout the procedure. Transmitter leads (TA11ETA-F10, Data Sciences International [DSI], St. Paul, MN) were placed into two 0.9 mm diameter holes positioned over the left and right cortical hemispheres (1–1.2 mm anterior to the lambda suture and 1.5 mm lateral to the midline). Care was taken not to perforate the dura so that skull encapsulation remained intact. Anchor screws (shaft diameter 1.16 mm, length 1.6 mm) were placed ≈2 mm anterior to the transmitter leads and both leads and screws were secured in place with dental cement. The transmitter was inserted into a subcutaneous pocket posterior to the neck. Following placement of the leads and transmitter, the incision was closed. The ability to close the wound completely is a significant advantage of wireless recording, as it greatly reduces infection risk and facilitates chronic recording. Mice were given 48 hours to recover before EEG recordings were begun and at least one week to recover before vehicle or CORT treatments were begun.

EEG and video recordings were performed in an isolated, sterile room equipped with the DSI wireless telemetry system running DATAQUEST A.R.T. (version 4.2) acquisition software (DSI). The room has the same light/dark cycle as the vivarium. The EEG signal was digitized at 250 Hz. A total of 9320 hours of video-EEG data was collected from eleven epileptic animals and 2906 hours was collected from five control animals. EEG data was analyzed by an experimenter unaware of treatment using Neuroscore software (version 2.0, DSI). Seizures were defined as a sudden onset of high amplitude (>2× background) activity with signal progression (a change in amplitude and frequency over the course of the event) and a duration greater than ten seconds (Fig. 1. B). Animals were also scored for periods of epileptiform activity that could not be strictly defined as seizures. Epileptiform activity was defined as periods of rhythmic spiking or bursting that lasted a minimum of 30 seconds. Activity included runs of spikes, polyspikes and sharp waves with a frequency of 0.5–2 Hz. In addition, 1–5 second bursts of paroxysmal high frequency (50–100 Hz) activity occurring every 5–20 seconds (Fig. 1, C) were scored when more than four bursts in a row occurred. The duration and frequency of epileptiform events was quantified for each animal in the 24 hour periods immediately preceding and following vehicle or CORT administration.

### Corticosterone treatment regimen

Epileptic animals were randomly assigned to either a vehicle-first (n = 7; Fig. 2, A) or CORT-first (n = 4; Fig. 5, A) regimen. In the vehicle-first regimen, animals were monitored for 1–2 weeks to determine baseline seizure frequency. After initial monitoring, animals were treated with vehicle for 5–7 days (7 µl propylene glycol/gram body weight 2×/day s.c.), followed by a 1–2 week recovery period and then 7 days of CORT treatment (3 mg/kg CORT in propylene glycol vehicle 2×/day s.c.). Two vehicle-first animals did not complete the full 7-day CORT treatment; one animal died after two days of treatment (Fig. 3, C) and one animal was treated for only three days as part of an initial pilot (Fig. 3, D). The CORT-first regimen was identical, except that the timing of CORT and vehicle treatments was reversed in order to control for the possibility that vehicle treatment and handling [71], [72] might alter later response to CORT. This also allowed us to control for the progressive nature of epilepsy in animal models, in which seizures become more frequent over time. CORT treatments were designed to mimic endogenous CORT levels observed during periods of chronic stress [73], [74]. Each injection leads to elevated CORT levels for less than 24 hours [75]–[77]. Throughout the entire protocol, video-EEG data was monitored continuously to ensure that no seizures would be missed.

### Corticosterone measurements

Levels of CORT were determined in a subgroup of control mice (n = 4) on the first days of vehicle and CORT treatment at three timepoints: 1) three minutes prior to the injection (baseline), 2) thirty minutes after vehicle or CORT injection and 3) two hours after after vehicle or CORT injection. Blood samples were collected from the tail [29] in EDTA-coated tubes. Samples were then centrifuged (3000 g, 15 min, −4°C) and plasma was isolated and stored at −20°C until the time of use. Plasma corticosterone levels were measured using a 125I radioimmunoassay (RIA) kit (MP Biomedicals Inc., Solon, OH). All samples were run in duplicate and in the same assay. Video-EEG was also recorded from this group of animals. Injections of vehicle and CORT were given between 8:30–9:30am in the morning and between 3:30–4:30pm in the afternoon.

### Statistical analysis

Statistical analyses were performed using SigmaPlot software (version 12.3). Statistical comparisons to examine seizures and epileptiform activity were made using a two-way ANOVA with animal and treatment (vehicle or CORT injection) as factors. Statistics were conducted on data normalized by rank transformation. A Holm-Sidak post-test was used to isolate significantly different groups. Other statistical tests used are indicated in the results. One-way ANOVA was run for seizure duration since not all animals had seizures during all treatment periods, so the data was disconnected and a two-way ANOVA was not possible. Measures are expressed as means ± SEM or medians (range) of the raw (untransformed) data. P values ≤0.05 were accepted as significant.
